# Radiation-Induced Carotid Artery Disease: Pathogenesis, Diagnosis and Management

**DOI:** 10.3390/diagnostics16060841

**Published:** 2026-03-12

**Authors:** Alfredo Mauriello, Adriana Correra, Anna Chiara Maratea, Giovanni Benfari, Federica Ilardi, Giuseppe Giugliano, Matteo Lisi, Alessandro Malagoli, Giulia Elena Mandoli, Maria Concetta Pastore, Simona Sperlongano, Vincenzo Russo, Matteo Cameli, Antonello D’Andrea

**Affiliations:** 1 S.C. Cardiology, Institute National Cancer, IRCCS, Foundation “G. Pascale”, Via M. Semmola 52, 80131 Naples, Italy; alfredo.mauriello93@libero.it; 2 Cardiology Department, Ospedali Riuniti University Hospital, Viale Pinto 1, 71122 Foggia, Italy; adrianacorrera@gmail.com; 3 Department of Cardiovascular Disease, ASL Napoli 1 Centro, Via Comunale del Principe, 13/a, 80145 Napoli, Italy; annachiara.maratea@gmail.com; 4 Section of Cardiology, Department of Medicine, University of Verona, P.le L.A. Scuro 10, 37100 Verona, Italy; giovanni.benfari@univr.it; 5 Department of Advanced Biomedical Sciences, Division of Cardiology, Federico II University Hospital, Via Pansini 5, 80131 Naples, Italy; federica.ilardi@unina.it (F.I.); giuseppe.giugliano@unina.it (G.G.); 6 Department of Cardiovascular Disease—AUSL Romagna, Division of Cardiology, Ospedale “S. Maria delle Croci”, Viale Randi 5, 48121 Ravenna, Italy; matteo.lisi@hotmail.it; 7 Division of Cardiology, Nephro-Cardiovascular Department, “Baggiovara” Hospital, Via P: Giardini 1355, 41100 Modena, Italy; ale.malagoli@gmail.com; 8 Department of Medical Biotechnologies, Division of Cardiology, University of Siena, Viale Bracci 16, 53100 Siena, Italy; giuliaelena.mandoli@unisi.it (G.E.M.); mariaconcetta.pastore@unisi.it (M.C.P.); matteo.cameli@unisi.it (M.C.); 9 Cardiology Unit, Department of Medical and Translational Sciences, University of Campania “Luigi Vanvitelli”, “I Policlinico” Hospital, Piazza Luigi Miraglia 2 snc, 80100 Naples, Italy; simona.sperlongano@unicampania.it; 10 Cardiology Unit, Department of Medical and Translational Sciences, University of Campania “Luigi Vanvitelli”, “V. Monaldi” Hospital, Via Leonardo Bianchi snc, 80131 Naples, Italy; vincenzorusso@unicampania.it; 11 Cardiology and Intensive Care Unit, Department of Cardiology, “Umberto I” Hospital, Via Alfonso De Nicola 1, 84014 Nocera Inferiore, Italy

**Keywords:** radiotherapy, carotid stenosis, carotid plaque, atherosclerosis, neck cancer

## Abstract

Patients undergoing radiotherapy (RT) for head and neck cancers (HNCs) face a significantly increased risk of developing carotid artery stenosis (CAS) and cerebrovascular disease (CVD). This condition, known as accelerated or radiation-induced carotid atherosclerosis, represents a long-term toxicity that profoundly impacts patients’ quality of life and survival. Pathogenesis is complex, involving mechanisms such as direct endothelial damage, oxidative stress, chronic inflammatory activation, peri-adventitial fibrosis, and the acceleration of pre-existing atherosclerotic processes. Despite this elevated risk, universal screening and treatment are not yet standardized across all international guidelines. This narrative review summarizes the epidemiology, pathological mechanisms, and clinical implications of accelerated radiation-induced carotid stenosis (RICS) after neck irradiation.

## 1. Introduction

**Figure 1:**
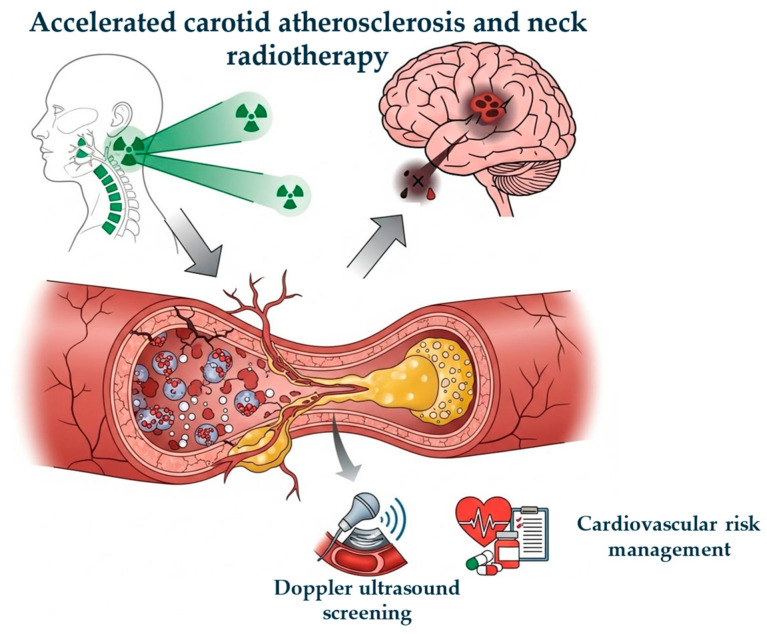
Relationship between neck radiotherapy and accelerated carotid atherosclerosis and their cardiovascular management.

Radiotherapy (RT) is a cornerstone of treatment for head and neck cancers (HNCs), contributing significantly to improved survival rates [1]. The increase in longevity in this population has led to greater attention on long-term toxicity, particularly that affecting the cardiovascular system [2]. Carotid arteries are inevitably exposed to significant doses of radiation during the irradiation of neck lymph nodes. Exposure to ionizing radiation is a recognized and well-documented risk factor for the development of carotid artery stenosis (CAS), known as radiation-induced carotid stenosis (RICS) [3]. An important aspect is the dose–response relationship: a significant effect for asymptomatic RICS has been observed even at low cumulative doses, such as 10 Gy [4]. Traditional risk factors such as smoking, arterial hypertension, dyslipidemia, diabetes mellitus (DM) and advanced age act synergistically with RT, although conventional cardiovascular risk prediction models tend to underestimate the risk in this population [5]. This condition is often referred to as accelerated atherosclerosis or radiation-induced vasculopathy [6]. The vascular damage induced by RT can manifest clinically many years after treatment completion. The most serious consequence is the increased risk of cerebrovascular events, such as ischemic stroke and transient ischemic attack (TIA) [7]. Although in the past research primarily focused on microvascular damage, it is now recognized that damage to large arteries, such as carotids, is of greater clinical relevance. This narrative review aims to comprehensively examine the epidemiology, pathophysiological mechanisms, clinical and dosimetric risk factors, diagnostic strategies, and current clinical and management implications of RICS after neck irradiation. Figure 1 represents the relationship between neck RT and accelerated carotid atherosclerosis and their cardiovascular management.

## 2. Materials and Methods

This manuscript presents a clinically focused narrative review of the current understanding of the link between carotid atherosclerosis and neck radiotherapy. Our central objective is to establish the pathophysiological connection between accelerated atherosclerosis and neck radiotherapy, and to explore potential preventive and therapeutic strategies. We offer a reasoned synthesis of the evidence, intentionally avoiding the formal structure of a systematic review or meta-analysis.

To ensure the selection of sources was authoritative and transparent, we employed a directed, yet non-exclusive, search strategy. A comprehensive literature search was executed through PubMed/MEDLINE and EMBASE for articles published between January 2010 and February 2026. The search utilized the following keywords and Boolean logic: “carotid” OR “carotid plaque” AND (“radiotherapy” OR “neck radiotherapy” OR “neck cancer). We restricted the results to English-language documents with available abstracts.

We prioritized the inclusion of prospective and retrospective clinical studies, randomized controlled trials (RCTs), meta-analyses, and registry reports that specifically addressed the relationship between carotid atherosclerosis and neck radiotherapy, excluding case reports, editorials, commentaries, comprehensive reviews, experimental pre-clinical studies lacking clear clinical relevance and conference abstracts without complete clinical data.

As this is a structured narrative synthesis and not a systematic review, no aggregated quantitative analysis was performed.

For observational research, we conducted a qualitative appraisal using the Joanna Briggs Institute (JBI) Critical Appraisal Checklists [8] and, where applicable, applied the Cochrane Risk-of-Bias framework to RCTs.

## 3. Epidemiology and Risk of Stenosis and Stroke

HNC survivors who have received RT are exposed to a remarkably elevated risk of RICS and cardiovascular disease (CVD) [4].

### 3.1. Prevalence and Cumulative Incidence

A meta-analysis including 1479 patients, with a primary endpoint to identify the prevalence, incidence, and degree of carotid stenosis in patients with a history of head and neck irradiation, found that the prevalence of RICS at 50% was 25% (95% confidence interval (CI): 19–32%, I^2^: 82.9%), during a follow-up of 2–13 years. The prevalence of stenosis >70% and carotid occlusion was 12% (95% CI, 7–17%, I^2^: 49%) and 4% (95% CI, 2–8%, I^2^: 56.3%), respectively, during a follow-up of 2–7 years [9].

Another meta-analysis, which included 35,160 patients, aims to analyze the effect of RT and update the risk of cardiovascular disease (CVD) following RT in HNC patients. It reported that the prevalence of RICS > 50% was 26% (95% CI, 22–31%; *p* < 0.0001) in HNC patients treated with RT [10]. Furthermore, an aggregate analysis of 13 case–control studies revealed a 7-fold higher risk of RT-related vasculopathy (RICS > 50%) compared to control groups [10]. When other risk factors were taken into account, having received any neck RT was associated with a 3.98-fold increased risk of cerebrovascular events (CVEs) [4].

The cumulative incidence of RICS increases significantly over time. According to a systematic review, the cumulative incidence of RICS > 50% at 12-, 24-, and 36-month post-RT was, respectively, 4% (CI 95%: 2–5%), 12% (CI 95%: 9–15%) and 21% (CI 95%: 9–36%) [9].

A retrospective study, including 628 HNC patients (80% male; mean age 61 years) treated with curative RT, during a follow-up to 10 years, aims to evaluate the cumulative incidences of CAS and CVD among HNC survivors after RT and whether CAS is associated with a RT dose–response effect. Authors concluded that the incidence of asymptomatic RICS at 10 years was 29.6% (95% CI, 23.9–35.5%), whereas for the symptomatic form, it was 10.1% (95% CI, 7.0–13.9%) [4]. Composite RICS, meaning asymptomatic or symptomatic, had an incidence at 10 years of 27.2% (CI 95%, 22.5–32.1) [4].

It is important to note that these risks are high even in the absence of traditional cardiovascular (CV) risk factors (Framingham risk factors). In a subgroup of patients without identifiable Framingham risk factors before RT, the cumulative incidence of asymptomatic RICS at 10 years was still 25.6% (CI 95%, 16.2–36%) [4].

### 3.2. Risk of Ischemic Stroke

Exposure to ionizing radiation to the neck has been shown to at least double the relative risk of TIA and stroke [11]. The cumulative incidence of ischemic cerebrovascular events (ICVEs) varies. One retrospective study, including 750 patients (75% male; mean age 63 years), aims to evaluate the event of an ischemic cerebrovascular accident or TIA in the anterior circulation after completion of radiotherapy. It reported a cumulative incidence of ICVEs of 4.6% at 5 years and 7.4% at 8 years [12]. In young patients (<60 years old) undergoing neck RT, the relative risk of ischemic stroke is significantly increased by 5.6 [13]. The risk appears to be more pronounced 5–10 years after completion of RT. The median time from treatment to stroke onset has been reported as 11 years in one study [14] and 10.9 years in another [15].

## 4. Pathogenesis of Radio-Induced Carotid Atherosclerosis

The exact mechanisms of RICS remain not fully defined but are generally attributed to a combination of accelerated atherosclerosis, intimal proliferation, medial necrosis, and peri-adventitial fibrosis [16].

### 4.1. Endothelial Damage and Acute Inflammation

Exposure to ionizing radiation leads to the formation of reactive oxygen species (ROS) and free radicals, causing deoxyribonucleic acid (DNA) damage and the oxidation of lipids and proteins [17]. Endothelial cells lining the tunica intima are susceptible to radiation. Acute endothelial damage leads to endothelial cell apoptosis and senescence, resulting in endothelial dysfunction [18]. Endothelial dysfunction is characterized by increased vascular permeability and impaired release of nitric oxide (NO), which hinders endothelium-dependent relaxation [19]. Simultaneously, there is an increase in the prothrombotic and procoagulative state, with greater production of von Willebrand factor (vWF) and platelet-activating factor, and reduced production of prostacyclin and thrombomodulin. This favors platelet adhesion and aggregation, predisposing to arterial thrombosis [19].

### 4.2. Chronic Inflammation and Fibrosis

In stable atherosclerotic plaques, smooth muscle cells (SMCs) play a protective role in the formation and maintenance of the fibrous cap [20]. Radiotherapy disrupts this homeostatic balance by inducing direct DNA damage within the SMC population, thereby precipitating cellular senescence or apoptosis [21,22]. Consequently, collagen production is attenuated, leading to progressive thinning of the fibrous cap [22]. Furthermore, residual SMCs may undergo a phenotypic switch, transitioning from a contractile, protective state to a pro-inflammatory, synthetic phenotype, thereby actively contributing to, rather than inhibiting, the pathological progression [23]. Macrophages and smooth muscle cells migrate into the arterial media in response to the accumulation of oxidized low-density lipoproteins (LDL) [24]. Within this environment, macrophages internalize oxidized low-density lipoproteins (oxLDL), differentiating into lipid-laden “foam cells” [6]. RT exacerbates local oxidative stress, accelerating LDL oxidation and promoting foam cell formation, thereby expanding the plaque’s necrotic core [25]. Radiation-activated macrophages further secrete elevated levels of matrix metalloproteinases (MMPs), proteolytic enzymes that degrade the collagen matrix [26]. The release of pro-inflammatory and pro-fibrotic cytokines is observed, including tumor necrosis factor-beta (TNF-beta) and transforming growth factor-beta (TGF-beta) [27]. TGF-beta is implicated explicitly in peri-adventitial tissue fibrosis, a process that contributes to vascular stiffness [27]. The clinical culmination of these synergistic processes is the development of a high-risk or “vulnerable” plaque: characterized by a thin fibrous cap overlying a lipid-rich, inflammation-heavy necrotic core, which significantly increases the propensity for rupture and subsequent acute vascular events [28]. This fibrotic process may also be responsible for the concentric increase in vascular wall thickness, which, in some studies, has been observed within 1 year and has not progressed significantly over time, unlike atherosclerotic stenosis, which worsens with time post-RT [27].

### 4.3. Distinguishing Morphological Characteristics

RT-induced vascular changes increase carotid intima-media thickness (CIMT), an early marker of atherosclerosis. Increased CIMT has been observed as early as 1–2 years after RT. A prospective study including 38 patients (60.5% male; mean age 59.1 years) found a significant increase in CIMT in the irradiated carotid artery compared to the non-irradiated one as early as 18 months (*p* = 0.014), a difference that persisted at 3 (*p* = 0.016) and 4-years post-RT (*p* < 0.0002) [29]. Additionally, another retrospective study, including 46 patients (48% male; mean age 52 years), aims to evaluate whether duration post-RT is associated with CIMT progression and further explores its association with mortality. reported a significant increase in CIMT and plaque score at 6 years post-RT compared to healthy controls, and CIMT was positively correlated with the duration of post-RT follow-up (*p* < 0.0001) [30].

Radio-induced atherosclerotic lesions exhibit distinct morphological and distributional characteristics compared to traditional atherosclerosis.

Location: radio-induced lesions involve the proximal common carotid artery (CCA) and the ICA, unlike typical atherosclerosis, which predominantly involves the carotid bifurcation and the proximal segment of the internal carotid artery (ICA). This more proximal location has implications for surgical management and screening procedures, as standard carotid Doppler ultrasound (DUS) might not fully evaluate this portion [31].

Plaque morphology: radio-induced stenosis tends to be longer and more segmental. Histologically, radio-induced plaques have been described as more fibrotic and less inflammatory compared to de novo plaques. Some studies suggest they might have a smaller necrotic core and less macrophage infiltration, indicating a more stable phenotype. However, another study has suggested a more ulcerated, mobile, and vulnerable phenotype [32].

## 5. Clinical and Dosimetric Risk Factors

Both oncology treatment-related factors and pre-existing CV factors modulate the risk of CAS and CVD.

### 5.1. Role of Traditional Risk Factors

Advanced age, smoking, systemic arterial hypertension, dyslipidemia, and DM are universally recognized as risk factors for atherosclerosis and have been extensively studied in the context of RICS [33]. Age is a significant predictor of asymptomatic CAS [33]. Smoking is strongly associated with an increased risk of CAS. Patients who developed stenosis >70% had significantly higher odds of being smokers [9]. Hyperlipidemia has been associated with the risk of asymptomatic CAS in several studies. Higher baseline total cholesterol levels have been linked to a greater incidence of post-RT atherosclerosis and a greater severity of stenosis [34]. DM and systemic arterial hypertension are commonly cited as factors that increase the risk of stroke and CAS in HNC patients undergoing RT. A prospective study, including 125 patients (97.6% male; 61.5 ± 9.7 years), aims to evaluate post-RT changes in the CCA using computed tomography (CT) and found that DM was independently associated with post-RT vascular calcification (24.3% vs. 9.1%, *p*  =  0.02) [35].

Traditional risk factors not only contribute to *de novo* CAS but are also suspected of accelerating the radio-induced atherosclerotic process.

### 5.2. Dosimetric Factors and Dose–Response Correlation

The RT dose is a critical determinant. Historically, doses as low as 0.5–10 Gy have been associated with an increased risk of late vascular events [36].

Historically, doses starting from 5 to 10 Gy have been associated with an increased risk of late vascular events. A detailed analysis of Carpentier et al. highlighted a significant association between asymptomatic RICS and the absolute carotid artery volume receiving >10 Gy (V10). Specifically, significant associations for asymptomatic RICS were found with V10, V20, V30, V40, V60 e V70 Gy. These results suggest that even low doses up to 10 Gy can increase the risk of asymptomatic RICS [4].

At the same time, von Aken et al. associated the absolute carotid V10 as a predictor of ischemic cerebrovascular events (HR 1.11; *p* < 0.001) [12]. However, a previous retrospective case–control study, including 43 patients, failed to identify an apparent dose–response effect [37]. This discrepancy could be due to limited follow-up, insufficient to capture late vascular events, or limitations in dosimetric analysis for lower doses. A significant association was found between the cumulative dose and the risk of carotid blowout, and the results support an existing dose constraint for the carotid arteries of 120 Gy for re-irradiation [38].

## 6. Clinical Manifestations and Diagnosis

### 6.1. Clinical Manifestations

The most severe clinical manifestation of RICS is ischemic stroke or TIA. Typical symptoms of extracranial carotid stenosis include amaurosis fugax, paresis, sensory disturbances, aphasia, and dysarthria [39]. The interval between irradiation and the onset of vascular symptoms ranges from a few months to two decades [14]. Patients are often relatively younger and present a lower incidence of atherosclerotic risk factors compared to non-irradiated patients, which makes RT-induced lesions a clinically distinct entity [40].

### 6.2. Diagnostic Tools and Early Detection

**Figure 2:**
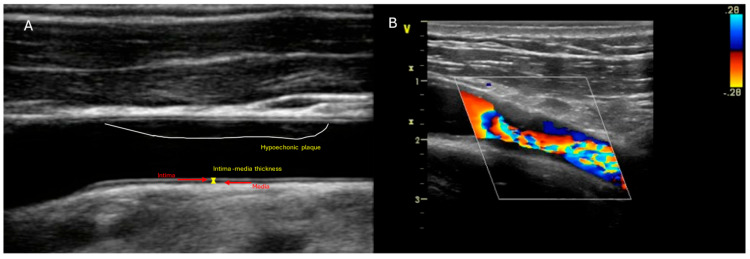
(**A**) represents the thickness of IMT and an echolucent plaque. (**B**) represents the presence of plaque in the internal carotid artery, with the aliasing phenomenon on color Doppler.

Early identification of RICS is fundamental for initiating preventive strategies. DUS is the most common imaging modality for evaluating RICS [41]. It is a non-invasive screening tool recommended by some guidelines. The measurement of CIMT has been widely used in the literature on HNC patients undergoing RT as an early marker of vascular damage [42]. A systematic review including 34 articles showed that two matched-control studies demonstrated significant increases in CIMT of 36% and 22% in irradiated carotid arteries compared with non-irradiated carotid arteries or healthy controls (*p* = 0.003 and <0.001, respectively) [7]. A prospective study of 125 patients (male 97%; mean age 62 years) with laryngeal cancer showed an increase in wall thickness and a decrease in lumen area several months after RT, changes that were not progressive but early. The rise in CIMT is positively correlated with the duration of post-RT follow-up (*p* = 0.02). In addition, 17% new carotid calcifications were observed within 4 years post-RT (*p* = 0.002) [35]. Figure 2 shows CIMT and the presence of plaque in the internal carotid artery, with the aliasing phenomenon on colour Doppler.

More sophisticated measures, such as the total plaque score (TPS), have proven useful. A retrospective study involving 217 patients (70% male; mean age 57 years) with stroke found that a TPS of 7 or higher was associated with an increased risk of RICS progression (*p* = 0.002) [43]. Contrast-enhanced ultrasound (CEUS) can detect intraplaque neovascularization, which was found to be increased in carotid atherosclerotic plaques related to previous neck RT [44].

Computerized tomography (CT) and magnetic resonance imaging (MRI) are not recommended for routine screening, but can be helpful for further evaluation and characterization of carotid disease.

CT provides excellent structural details, including assessment of the aortic arch, which is essential for the surgical planning of more proximal lesions. The disadvantage is exposure to radiation and iodinated contrast medium [45]. A retrospective cohort study assessed 44 patients (male 86.4%) with HNC who underwent RT [46] showed that low-density plaque was a critical predictor of accelerated radiation-induced carotid stenosis (before low-density plaque detection to 4.7%/year (interquartile range (IQR), 3.4–8.1%), afterwards (*p* < 0.001), with a marked acceleration to 14.8%/year (IQR, 9.6–24.0%). This study proposes a novel biphasic progression model with a slow phase before low-density plaque detection and a rapid phase thereafter. These findings support intensified surveillance, such as annual CT scans, and timely interventions to prevent occlusion in high-risk patients.

The review of available CT scans, often performed for oncological surveillance, to detect carotid calcifications, is recommended as a tool to identify asymptomatic atherosclerosis [35].

MRI offers an alternative without ionizing radiation and contrast, although it is more expensive and time-consuming [47].

Recently, the study of multiple positron emission tomography (PET) radiotracers has taken on an important role, potentially providing novel diagnostic insights into carotid atherosclerotic plaque and improving the identification of vulnerable carotid atherosclerotic plaque. A retrospective study including 181 patients (71.8% male; mean age 70.2 years), aims to evaluate 5-year recurrent ipsilateral ischemic stroke after PET imaging, showed that plaque inflammation-related ^18^FDG uptake improved the identification of 5-year recurrent ipsilateral ischemic stroke (adjusted HR 2.73 per 1-point increase, 95% CI 1.52–4.90, *p* = 0.001) [48].

Although standard imaging, such as DUS, is the most common modality for evaluating CAS, advanced techniques are increasingly relevant for distinguishing stable fibrotic plaques from more vulnerable phenotypes, such as ulcerated or mobile plaques, often observed following neck radiotherapy [49].

## 7. Clinical Implications and Surveillance Strategies

### 7.1. Need for Targeted Screening

Routine carotid DUS screening should be performed, given the high risk of RICS and CVD in HNC survivors who undergo RT. The Society for Vascular Surgery (SVS) guidelines suggest that screening for asymptomatic RICS is cost-effective when the prevalence of significant stenosis is >20%. Since the aggregated prevalence in RT-treated patients vastly exceeds this threshold, this provides a strong justification for screening in this high-risk population [5].

### 7.2. Inadequacy of Traditional Risk Models

It is essential to recognize that conventional cardiovascular risk prediction tools, such as the Framingham [50], QStroke [51], and QRISK-2 [52] scores tend to significantly underestimate the risk of ischemic stroke in HNC patients treated with RT. RT represents an independent and significant risk factor. This underscores the need to inform both survivors and treating physicians about the elevated risk and the importance of CV prevention strategies [53].

### 7.3. Recommendations for Screening and Surveillance

International guidelines are not yet fully aligned on routine screening.

International Cardio-Oncology Society (ICOS) [54]: recommends auscultation for carotid bruits during routine physical examination. It recommends carotid DUS for screening in asymptomatic individuals with atherosclerotic plaque. Initial DUS can be considered as early as 1-year post-RT in high-risk patients (defined by radiation dose and CV risk), with follow-up every 3–5 years to guide preventive therapy. It also suggests reviewing available CT scans for carotid calcifications.

American Head and Neck Society (AHNS) [55]: suggests considering carotid DUS every 2–5 years after completion of RT.

National Comprehensive Cancer Network (NCCN) [56] and American Society of Clinical Oncology (ASCO) [57]: acknowledge the risk of RICS and stroke, but do not provide specific screening recommendations due to the presumed insufficient evidence that routine screening influences outcomes.

Table 1 summarizes recommendation from several guidelines regarding screening and surveillance of CAS post-RT.

It is essential that clinicians, including radiation oncologists, discuss the increased long-term risk of RICS and stroke with patients during informed consent. The first baseline assessment should be performed 1–2 years after completing RT. In the absence of plaques, it is usually repeated every 3–5 years (or more frequently in the presence of other cardiovascular risk factors).

## 8. Management of Radio-Induced Carotid Stenosis

Management of RICS is based on aggressive modification of CV risk factors and, for symptomatic cases or those with severe stenosis, on revascularization intervention

### 8.1. Medical Prevention Therapy

The most robust strategy is the optimal management of modifiable risk factors.

The use of statins is a therapy with the most substantial evidence for CV prevention in this population. Statins reduce RT-related carotid artery damage by lowering cholesterol levels. Furthermore, they exert pleiotropic effects, including limiting post-irradiation endothelial activation and reducing inflammatory and thrombotic responses. Retrospective studies [58,59,60] have shown a significantly reduced risk of stroke for cancer patients undergoing RT to the head, neck, or chest who were taking statins. In patients with intermediate or high 10-year atherosclerotic cardiovascular disease risk, the initiation of statin therapy is recommended [61]. In patients with lower risk (<7.5%) but with prior neck RT, statin therapy should be considered, especially if there is evidence of carotid plaque or calcification (detected on CT or DUS) [62].

The use of antiplatelet therapy, such as aspirin, for the primary prevention of stroke in RT survivors warrants further study. Patients with documented RICS or carotid plaques should initiate statin therapy for the primary prevention of ischemic stroke, in line with general guidelines [58].

### 8.2. Revascularization

For symptomatic patients or those with asymptomatic CAS >70%, revascularization interventions are indicated. In asymptomatic CAS, beyond the 70% stenosis threshold, clinicians should evaluate several critical “high-risk” features that suggest medical therapy alone may be insufficient.

Microembolic signals (MES): detected via transcranial Doppler (TCD), the presence of spontaneous emboli (1 ± 3) in the middle cerebral artery is a strong predictor of imminent stroke risk in asymptomatic patients [63].

Progression velocity: a rapid increase in the degree of stenosis is a major indication for RICS, as radiation-induced disease can follow a “biphasic” or accelerated trajectory [46].

Silent infarcts on MRI: the presence of small, old embolic “silent” infarcts in the ipsilateral hemisphere, detected on fluid attenuated inversion recovery (FLAIR) or diffusion-weighted (DW) MRI, indicates that the lesion is biologically active despite the lack of clinical symptoms [64].

Plaque echolucency: hypoechoic plaques are associated with greater instability and higher stroke risk than heavily calcified plaques [65].

The two main procedures are carotid endarterectomy (CEA) and carotid artery stenting [56].

CEA in irradiated fields poses significant challenges. RT-induced fibrosis and neck tissue scarring (hostile neck) make dissection more difficult and increase the risk of complications, particularly cranial nerve injury (CNI) [66]. Carotid artery stenting is often preferred in these cases because it avoids dissection in the fibrotic field [66].

The literature directly comparing CEA and carotid artery stenting for radio-induced stenosis is heterogeneous, but a meta-analysis including 7235 patients provides an updated picture. The rate of CNI (transient or persistent) was significantly higher in the CEA group compared to the carotid artery stenting group (RR: 6.03; IC 95%: 1.63–22.22; *p* = 0.007). Meta-regression analysis suggested that the carotid artery stenting group had significantly higher odds of stroke in both the short-term and long-term compared to the CEA group (short-term: carotid artery stenting 1.12% vs. CEA < 0.01%; long-term: carotid artery stenting 3.76% vs. CEA 0.9%). Restenosis or occlusion was more frequent in the carotid artery stenting group (*p*< 0.001) [67].

In general, although carotid artery stenting carries a lower risk of immediate CNI, updated evidence suggests that CEA may yield better long-term outcomes for these patients in terms of stroke prevention and restenosis. However, the choice must be individualized, considering the nature of the lesion and the condition of the neck.

## 9. Conclusions and Future Perspectives

Neck radiotherapy for head and neck cancers is a significant risk factor for the development of RICS, with rates of 50% stenosis reaching 25–30% in the long term. The risk of ischemic cerebrovascular events is at least twice as high in this population. The pathological mechanisms involve acute endothelial damage, chronic inflammation, and peri-adventitial fibrosis, culminating in RICS, often with distinct morphological characteristics. The dose–response relationship is well established, with the risk of asymptomatic RICS increasing even at doses as low as 10 Gy. Use of emerging radiotherapy modalities, such as heavy-ion therapy, proton therapy, boron neutron capture therapy, and γ-radiosurgery, may differ in their effects on vascular diseases, including carotid atherosclerosis; further research is needed. Aggressive management and optimization of cardiovascular risk factors are recommended, in close collaboration with primary care physicians, as traditional risk models underestimate the true incidence of ischemic stroke in these patients. The use of statins is strongly supported in prevention. Although screening guidelines are not universal, some societies suggest considering periodic carotid DUS, especially in high-risk patients, starting at 1–2 years after RT. For significant stenosis, the choice between CEA and carotid artery stenting must balance the risk of CNI (which is higher with CEA) and the risk of long-term stroke/restenosis (which may be higher with carotid artery stenting). Evidence suggests that CEA might offer superior long-term stroke prevention, but carotid artery stenting remains a valuable option for the “hostile neck”. Future studies are urgently needed, extensive prospective studies and RCTs, to: refine risk prediction models specific to post-RT HNC patients, integrating dosimetric and clinical factors; identify the optimal imaging modality for predicting future risk; evaluate the role and efficacy of preventive drugs in RCTs aimed at reducing radio-induced vasculopathy; and determine optimal screening intervals and the impact of modern RT techniques on long-term outcomes. These efforts are essential to develop evidence-based guidelines that enhance the management and quality of life for radiation therapy survivors.

## Figures and Tables

**Table 1:** Recommendation of several guidelines regarding screening and surveillance of carotid stenosis post-RT.

Organization/Guideline	Screening Recommendation
International Cardio-Oncology Society (ICOS) [54]	DUS to be considered at 1 year post-RT in high-risk patients with follow-up every 3–5 years to guide prevention. Review of CT scans for carotid calcifications. Auscultation for carotid bruits.
American Head and Neck Society (AHNS) [55]	Consider Carotid DUS every 2–5 years after completion of RT.
National Comprehensive Cancer Network (NCCN) [56]	Consider Carotid DUS every 2–5 years after completion of RT.
American Society of Clinical Oncology (ASCO) [57]	Acknowledges the risk of CAS. No specific recommendation for screening.
European Head and Neck Society (EHNS)	No discussion on CAS or stroke in the guideline.

CAS: carotid artery stenosis; CT: computerized tomography; DUS: Doppler ultrasound; RT: radiotherapy.

## Data Availability

No new data were created or analyzed in this study.
